# Combining the Sterile Insect Technique with the Incompatible Insect Technique: I-Impact of *Wolbachia* Infection on the Fitness of Triple- and Double-Infected Strains of *Aedes albopictus*

**DOI:** 10.1371/journal.pone.0121126

**Published:** 2015-04-07

**Authors:** Dongjing Zhang, Xiaoying Zheng, Zhiyong Xi, Kostas Bourtzis, Jeremie R. L. Gilles

**Affiliations:** 1 Insect Pest Control Laboratory, Joint FAO/IAEA Division of Nuclear Techniques in Food and Agriculture, Vienna, Austria; 2 Sun Yat-sen University—Michigan State University Joint Center of Vector Control for Tropical Diseases, Zhongshan School of Medicine, Guangzhou, China; 3 Department of Microbiology and Molecular Genetics, Michigan State University, East Lansing, Michigan, United States of America; University of Poitiers, FRANCE

## Abstract

The mosquito species *Aedes albopictus* is a major vector of the human diseases dengue and chikungunya. Due to the lack of efficient and sustainable methods to control this mosquito species, there is an increasing interest in developing and applying the sterile insect technique (SIT) and the incompatible insect technique (IIT), separately or in combination, as population suppression approaches. *Ae*. *albopictus* is naturally double-infected with two *Wolbachia* strains, *w*AlbA and *w*AlbB. A new triple *Wolbachia*-infected strain (i.e., a strain infected with *w*AlbA, *w*AlbB, and *w*Pip), known as HC and expressing strong cytoplasmic incompatibility (CI) in appropriate matings, was recently developed. In the present study, we compared several fitness traits of three *Ae*. *albopictus* strains (triple-infected, double-infected and uninfected), all of which were of the same genetic background (“Guangzhou City, China”) and were reared under the same conditions. Investigation of egg-hatching rate, survival of pupae and adults, sex ratio, duration of larval stages (development time from L_1_ to pupation), time to emergence (development time from L_1_ to adult emergence), wing length, female fecundity and adult longevity indicated that the presence of *Wolbachia* had only a minimal effect on host fitness. Based on this evidence, the HC strain is currently under consideration for mass rearing and application in a combined SIT-IIT strategy to control natural populations of *Ae*. *albopictus* in mainland China.

## Introduction

*Aedes albopictus*, one of the most invasive mosquito species [1], is the primary vector of the chikungunya virus and secondary vector of dengue viruses. However, in some areas, like mainland China, this mosquito plays a primary role in the transmission of dengue [2], with more than 43,000 cases reported in Guangdong in 2014 (WHO Dengue situation update 453). With no vaccine available, controlling the vector population is the only way of preventing or limiting the transmission of the chikungunya and dengue viruses. For several decades, vector control methods have mainly relied on the extensive use of insecticides, and this has resulted in an increased appearance of insecticide resistance in *Ae*. *albopictus* populations [3]. Furthermore, insecticides have a negative impact on non-target insect populations and can cause major toxicological effects on human health and the environment [4–6]. In addition to insecticide use, source reduction (destruction of mosquito oviposition sites) is another common approach to control, but is not sustainable, especially on a large scale [7]. Consequently, additional control methods are needed for the control of *Ae*. *albopictus*.

There has recently been an increased interest towards the development and application of genetic control methods such as the sterile insect technique (SIT) and the incompatible insect technique (IIT) to control mosquitoes including *Ae*. *albopictus*. The main difference between the SIT and the IIT lies in the sterilization means: gamma or x-ray irradiation is used in the former instance, an infection with *Wolbachia* in the latter. The sterile insect technique (SIT) is a species-specific and environmentally-friendly method which includes the mass-rearing of the target species, sterilization and inundative releases of the male insects into the target population. The released sterile males mate with and inseminate wild females, and through sequential releases the target population is suppressed [7]. Several successful SIT programs are in progress or have been concluded, including the eradication programs against the New World screwworm, *Cochliomyia hominivorax* Coquel in North America [8] and the tsetse fly, *Glossinaausteni* [9] on the island of Unguja.

*Wolbachia* is a maternally-transmitted obligate intracellular *Alphaproteobacterium* which is widespread among insect species [10, 11]. Although mutualistic nutrition as well as other beneficial associations have been reported [12–15], the presence of *Wolbachia* in insect hosts has been commonly associated with reproductive phenotypes such as male killing, feminization, parthenogenesis and cytoplasmic incompatibility (CI) [10, 11]. In diplo-diploid species, CI usually manifests as embryonic mortality resulting from mating between a *Wolbachia*-infected male and a female which is either uninfected or infected with a different *Wolbachia* strain [16, 17]. The IIT is based on the mechanism of *Wolbachia*-induced CI and relies on the repeated releases of incompatible males which will mate with wild type females leading to the gradual suppression of the target population [18–20]. The first successful application of IIT was achieved in Burma where the target population of the filariasis vector *Culex pipiens* was almost eliminated [21]. Feasibility studies for the use of IIT, with or without irradiation, to control populations of the mosquito species *Ae*. *albopictus* [22], *Ae*. *polynesiensis* [23], *Cx*. *pipienspallens* [24] and *An*. *stephensi* [25] have provided encouraging results both in the laboratory and in the field.

*Ae*. *albopictus* is naturally single (*w*AlbA or *w*AlbB) or double-infected (*w*AlbA and *w*AlbB) with *Wolbachia* [26–29]. Double-infected *Ae*. *albopictus* males have been shown to express strong CI in crosses with either uninfected or single-infected females [28]. Several artificially-infected lines have also been established via embryonic microinjections mainly with the aim of using them for the control of natural populations of *Ae*. *albopictus*. Four different *Wolbachia* strains (*w*Mel, *w*MelPop, *w*Ri and *w*Pip) have been transferred to *Ae*. *albopictus*, establishing single- or triple-infected lineswith diverse CI patterns (see Table 1 in Bourtzis et al. 2014) [7, 22, 30–34]. The effect of *Wolbachia* infection on the fitness of the naturally double-infected as well as the transinfected lines has been investigated. However, in most cases, this analysis has not been done in a thorough and systematic manner, failing to take into consideration the traits and factors important for mass rearing and large scale applications including the genetic background of the target natural population and its fitness in the field, especially mating competitiveness, flight ability and longevity [28, 31, 33, 35–37].

**Table 1:** The locations of *Aedes albopictus* larvae collected in the field of Guangzhou.

**Number**	**Distracts**	**Latitude**	**Longitude**
1	Conghua	23°41'37.72" N	113°52'35.09" E
2	Conghua	23°29'50.48" N	113°33'6.45" E
3	Baiyun	23°16'18.71" N	113°13'27.56" E
4	Baiyun	23°13'35.07" N	113°16'42.64" E
5	Baiyun	23°11'2.37" N	113°14'51.52" E
6	Baiyun	23°10'8.09" N	113°15'40.25" E
7	Tianhe	23° 9'15.51" N	113°19'30.83" E
8	Tianhe	23° 7'33.76" N	113°19'6.00" E
9	Liwan	23° 7'14.47" N	113°14'0.24" E
10	Liwan	23° 7'26.99" N	113°13'56.89" E
11	Yuexiu	23° 9'3.17" N	113°16'54.92" E
12	Yuexiu	23° 7'13.43" N	113°15'55.67" E
13	Yuexiu	23° 7'12.84" N	113°15'34.88" E
14	Haizhu	23° 5'55.69" N	113°15'58.89" E
15	Haizhu	23° 5'56.10" N	113°16'30.73" E
16	Haizhu	23° 5'45.05" N	113°15'38.92" E
17	Haizhu	23° 5'25.78" N	113°16'17.09" E
18	Panyu	22°56'18.87" N	113°21'12.34" E
19	Panyu	22°55'10.15" N	113°20'6.59" E
20	Nansha	22°44'8.50" N	113°28'59.11" E

Recently, a triple-infected (*w*AlbA, *w*AlbB and *w*Pip) *Ae*.*albopictus* HC strain has been established which expresses strong CI against double-infected or uninfected strains with the same genetic background (Xi et al., unpublished data). This line could be used for the control of natural populations of *Ae*. *albopictus* by IIT, SIT or using a combination of irradiation- and *Wolbachia*-induced sterility which, in the absence of a perfect sexing system, would allow male-only releases [7]. However, the mass rearing and quality control analysis of the strain which will be used to introduce sterility in nature are prerequisites prior to large scale implementation of any of these approaches [7]. In the present study, we performed a comparative analysis of the impact of *Wolbachia* infection on several fitness traits of three *Ae*. *albopictus* strains with a high similarity of genetic background but with different *Wolbachia* infection status (triple-infected, double-infected and uninfected). Our results are discussed in view of using the triple-infectedstrain as a tool to suppress *Ae*. *albopictus* populations in pilot sites in mainland China.

## Methods

### Ethics Statement

The *Ae*. *albopictus* larvae were collected by the Center of Disease Control and Prevention of Guangzhou in strict accordance with the principles of the institutional and national Committees of Animal Use and Protection. No specific permissions were required for *Ae*. *albopictus* studies in Guangzhou. The 20 locations, where the larvae were collected, were not private or protected areas and did not involve endangered or protected species (Table 1). The blood used for routine blood-feeding was collected in Vienna, Austria during routine slaughtering of pigs or cowsin a nationally authorized abattoir, conducted at the highest possible standards strictly following EU laws and regulations.

### Mosquito strains and rearing conditions

Three *Wolbachia*-infected strains of *Ae*. *albopictus* were used in the present study: the double-infected strain of GUA from Guangzhou City, China (*w*AlbA and *w*AlbB), the HC strain (triple-infected with *w*AlbA, *w*AlbB and *w*Pip) and the *Wolbachia*-free aposymbiotic line (GT) strain. The GUA strain was established from 2,000 mosquito larvae collected from 20 different districts of the Guangzhou metropolitan area in March 2013 (100 larvae collected per district). The strain was maintained at a high population size (several thousand mosquitoes) in the laboratory for ten generations [eight generations in Guangzhou, China and two generations in the Insect Pest Control Laboratory (IPCL), FAO/IAEA Joint Division, Seibersdorf, Austria] before being used for any experimental work. The triple *Wolbachia*-infected HC strain was generated by the transfer of the *w*Pip strain (originally from *Culex pipiens molestus*), through embryonic cytoplasmic injections, into the *Ae*. *albopictus* Houston strain. A single triple-infected female (*w*AlbA, *w*AlbB and *w*Pip) was used for the establishment of the HC line. Until submission of thismanuscript, the infection has been stably maintained for at least three years. The transinfected line induces almost complete unidirectional CI toward the wild type Houston strain (Xi, unpublished data).

HC females were outcrossed with GUA males for 6 consecutive generations. The outcrossing involved 100 virgin triple-infected females with 100 virgin GUA males in each generation in order to establish the HC line, with similar genetic background as the GUA strain. The strain was maintained at high population size (several thousand mosquitoes at each generation) in the laboratory for an additional two generations before being used for experimental work. The GT line was developed in Guangzhou by rearing several thousand adult mosquitoes with 10% sugar solution containing tetracycline (1.0 mg/ml) for five consecutive generations. The strain was maintained at high population size (several thousand mosquitoes) in the laboratory for two generations in 10% sugar solution without tetracycline before being used for any experimental work. The successful removal of *Wolbachia* and the maintenance of the symbiont-free status of the line was monitored by a standard *wsp* gene-based PCR assay using the primers 81F and 691R [26]. Since their establishment in the IPCL (September 2013), the GUA, HC and GT strains have been maintained in a climate-controlled room at 27 ± 1°C, 80 ± 10% RH, and a photoperiod of 12:12 (L:D) h. Taken together, the impact of *Wolbachia* infection type on the fitness of the three *Ae*. *albopictus* strains was assessed in generation G10 for the GUA strain, G8 for the HC strain and G7 for the GT strain.

Larvae were reared in plastic trays (40×29×8 cm) at a density of 3,000 first-instar larvae (L_1_) per tray that contained 1 liter of deionized water and were fed on IAEA2 diet regime [38] with the following minor modifications. The diet (7.5%) used for our experiments was made of 26.26g (35%) Bovine Liver Powder (MP Biomedicals, Santa Ana, CA), 37.5g (50%) Tuna Meal (T.C. Union Agrotech, Thailand)and 11.24g (15%) Brewer Yeast(Sigma Aldrich Inc., St. Louis, MO) mixed in 1 liter of deionized water. Pupae were collected and placed in small plastic cups inside a clean adult cage for emergence. Adults were kept in standard 30×30×30 cm plastic cages (BugDorm 1; MegaView, Taichung, Taiwan) and continuously supplied with a 10% sugar solution. Bovine or porcine blood was provided to female mosquitoes (4 to 5 days old) twice a week and moist oviposition papers (white crêpe paper IF C140, Industrial FiltroS.r.l., ColognoMonzese, Italy) were put into cage 48 hours after blood-feeding for collection of eggs. Forty-eight hours later, the filter paper was removed from the cage and allowed to dry for 24 hours, then the egg paper was placed in a plastic bag and stored in a sealed box in a climate-controlled room (27 ± 1°C, 80 ± 10% RH).

### Immature development of the *Ae*. *albopictus* HC, GUA and GT strains

For each strain, 3 replicates of 200–300 7 day-old eggs were transferred into a hatching solution made of 700 ml deionized water containing 0.25 g Nutrient Broth and 0.05 g Brewer Yeast at 27 ± 1°C. The parental fertility was measured by counting the number of eggs hatched from the total egg number laid per female, observed under the stereomicroscope.

Larval development and survival were determined by transferring 200 larvae (< 16h old) of each strain to a 10 cm diameter×10 cm plastic cylinder filled with 66 ml deionized water (around 3 L_1_/ml). Larval diet (7.5%) was provided on a daily basisas follows: 0.7 ml (0.26mg/larvae), 1.2 ml (0.45mg/larvae), 1.7 ml (0.64mg/larvae), 2.5 ml (0.94mg/larvae), 3.0 ml (1.1mg/larvae), and 2.5 ml(0.94mg/larvae), on days 1 to 6, respectively. All containers were observed daily at 9:00 am, 12:00 am and 15:00 pm and pupae were removed and transferred into plastic tubes (2 cm diameter×10 cm height) for emergence (no more than 10 pupae per tube). Each tube was filled with 20 ml of deionized water and covered with a sponge plug. Adult emergence was also recorded daily at 9:00 am, 12:00 am and 15:00 pm, and sex was determined. Three replicates of 200 larvae each were performed per strain.

Time to pupation and time to emergence were both recorded as the time required for the development of L_1_ to pupa and L_1_ to adult stages, respectively. Survival to pupation and survival to adult emergence were calculated as the proportion of larvae that survived from L_1_ to pupal stage and from L_1_ to adult stage, respectively.

### Wing length

To assess adult size, the left wing of 20 males and 20 females from each strain was collected for viewing under a stereomicroscope. The wing length was determined by measuring the distance from the distal edge of the alula to the end of the radius vein (excluding fringe scales). A digital image of the wing was made using a CC-12 camera mounted on a stereomicroscope and the actual measurement was performed using the analysis B software (Olympus Soft Imaging Solutions GmbH, Munster, Germany). Three replicates of 20 males and 20 females each were conducted per strain.

### Adult longevity and female fecundity

To assess adult longevity, 50 newly emerged males (maintained in the absence of females) and 30 newly emerged females (maintained together with 30 males) were placed in 30×30×30 cm plastic cages with constant access to 10% sugar solution. Female longevity was determinedunder two feeding regimes: (a) 10% sugar solution and (b) 10% sugar solution plus a blood meal on the 5^th^ and 25^th^ day. Oviposition cups were provided to the gravid females 48 hours after blood-feeding. Dead adults were removed and recorded daily until all females died. Three replicates were performed per strain.

To assess female fecundity, 25 newly emerged males and 25 newly emerged females (ratio 1:1) were placed in insect cages as previously described. Ten day old females were blood-fed and transferred into individual plastic tubes with moist filter paper. Fecundity was determined by counting the total number of eggs laid per female. Three replicates were conducted for each strain.

### Statistical analysis

The statistical analysis was conducted using the SPSS 13.0 software and GraphPad Prism 6.0 software. Egg hatch rate, sex ratio (expressed as proportion of females per total number of adults), survival to pupal stage (from L_1_ to pupa) and survival to adult stage (from L_1_ to adult) data were arcsine transformed. Normalcy of the data was assessed by the D’Agostino-Pearson omnibus normality test of the GraphPad Prism 6.0 software. Comparisons between strains were performed by one-way analysis of variance (ANOVA) and Tukey’s post hoc tests (P<0.05). One-way ANOVA and Tukey’s post hoc tests were also used to compare the mean time topupation and the mean time to emergence, wing length and fecundity. Independent *t*-test was used to assess potential differences according to sex observed on the mean time to pupation, the mean time to adult emergence and wing length within the same strain. Kaplan-Meier survival analyses (P<0.05) were conducted to determine differences in adult longevity between strains.

## Results

The artificially triple-infected (*w*AlbA, *w*AlbB, *w*Pip) HC strain produced by backcrossing and the *Wolbachia*-free GT strain produced by tetracycline treatment were considered to have a highly similar genetic background to the naturally double-infected (*w*AlbA, *w*AlbB) *Ae*. *albopictus* GUA strain originating from the Guangzhou metropolitan area in China.

### Immature development of the *Ae*. *albopictus* HC, GUA and GT strains

Under the same rearing conditions, there was no difference in the mean hatch rate of the eggs of the *Ae*. *albopictus* HC, GUA and GT strains (F = 0.28, df = 2, P>0.05) which ranged from 82% to 85% (Table 2). Survival both to pupation and to adult emergence did not differ significantly among the HC, GUA and GT strains (F = 3.82, df = 2, P>0.05 and F = 3.82, df = 2, P>0.05, respectively) (Table 2). In addition, no significant difference in adult sex ratio was observed between the three *Ae*. *albopictus* strains (F = 0.55, df = 2, P>0.05) (Table 2).

**Table 2:** Egg hatching rate, survivorship, and sex ratio (Mean ± SE) of the *Ae*. *albopictus* HC, GUA and GT strains.

**Strain**	**Egg hatching rate (%)**	**Survival rate fromL**_1_ **to pupa (%)**		**Survival rate fromL**_1_ **to adult (%)**		**Sex ratio** ^a^ **(%)**
HC	83.2 ± 1.7 a	90.3 ± 1.1 a	89.3 ± 0.2 a		53.9± 0.5 a	
GUA	85.6 ± 3.3 a	94.5 ± 1.8 a	93.7 ± 1.9 a		52.4± 2.8 a	
GT	82.0 ± 4.7 a	89.4 ± 0.5 a	88.5 ± 1.0 a		50.2± 3.3 a	

^a^ Sex ratio was calculated as the proportion of females out of the total number of adults

Within a column, values followed by different lowercase letters were statistically different; ANOVA was performed for egg hatching rate, survivorship, and sex ratio analysis (P<0.05).

As shown in Table 3, the triple-infected HC strain exhibited faster development from first instar larvae to pupation and from first instar larvae to adult emergence compared to the double-infected GUA and the uninfected GT strains for both males (F = 21.86, df = 2, P<0.05; F = 22.65, df = 2, P<0.05, respectively) and females (F = 5.66, df = 2, P<0.05; F = 7.57, df = 2, P<0.05, respectively). For all the strains, the time both to pupation and to adult emergencewas always about one day shorter for males than females (HC, t = 12.83, df = 533.33, P<0.05; t = 12.88, df = 522.34, P<0.05, respectively; GUA, t = 13.25, df = 572.90, P<0.05; t = 14.13, df = 563.21, P<0.05, respectively; and GT, t = 7.76, df = 509.98, P<0.05; t = 7.76, df = 499, P<0.05, respectively) (Table 3).

**Table 3:** Developmental time (Mean ± SE) from L_1_ to pupa formation and from L_1_ to adult emergence of the *Ae*.*albopictus* HC, GUA and GT strains.

**Strain**	**Mean time to pupation (d)**		**Mean time to adult emergence (d)**	
	**Male**	**Female**	**Male**	**Female**
HC	4.82 ± 0.06 a	6.06 ± 0.08 c	6.86 ± 0.06 a	8.08 ± 0.07 c
GUA	5.29 ± 0.06 b	6.35 ± 0.06 d	7.34 ± 0.05 b	8.42 ± 0.05 d
GT	5.45 ± 0.09 b	6.36 ± 0.08 d	7.49 ± 0.09 b	8.38 ± 0.08 d

Within a column, values followed by different lowercase letters were statistically different (P<0.05) using ANOVA and Tukey’s post hoc test analysis. Independent *t*-test was used to compare potential differences in the mean time to pupation and to emergence of males and females among the three strains studied (P<0.05).

### Wing length

**Fig 1:**
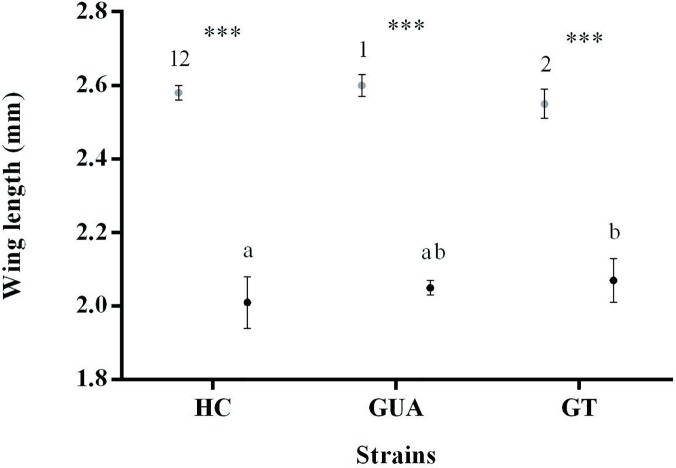
Box plots of wing length measurements (Mean ±SD) for females (graypoint) and males (black point). Boxes with the same number or letter were not significantly different betweeneach line, P<0.05 (Tukey’s post-hoc test). Asterisks (***) indicate a significant difference between male and femalewithin the same strain, P<0.05 (Independent *t*-test).

In each of the three strains, females had significantly longer wings than males (HC:t = 34.81, df = 118, P<0.05; GUA: t = 33.4, df = 118, P<0.05; GT: t = 25.08, df = 118, P<0.05) (Fig 1). Sex-dependent differences were observed in wing length between the HC, GUA and GT strains. The wings of GUA females were significantly longer than those of GT females (F = 4.85, df = 2, P<0.05) (Fig 1). Similarly, the wings of GT males were significantly longer than those of HC males (F = 4.63, df = 2, P<0.05) (Fig 1).

### Female fecundity and adult longevity

**Fig 2:**
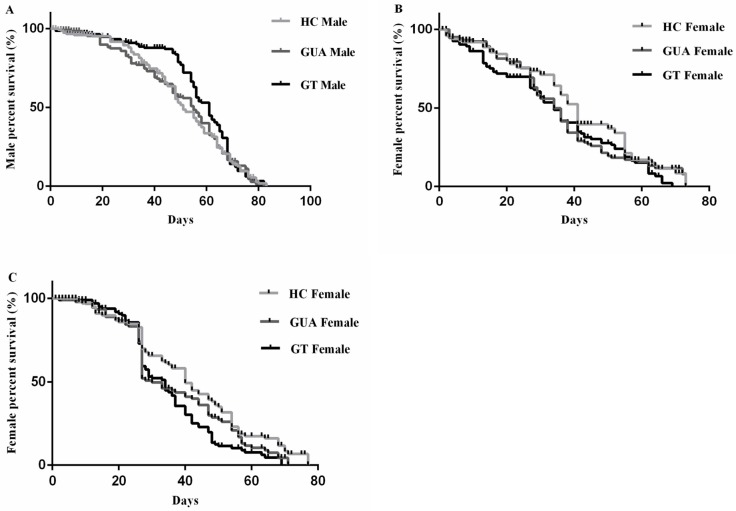
Adult survival curves for the *Ae*. *albopictus*HC, GUA and GT strains. Day number indicates time post-emergence. Kaplan-Meier curves were used to estimate the adultsurvivor function. A: Males only and fed on sugar; B: Females together with males and fed on sugar only; C: Females together with malesandfed on sugar and blood.

No significant difference was observed in the mean number of eggs laid per female between the three strains (F = 0.39, df = 2, P>0.05) (Table 4). With regards to male longevity, no significant difference was observed between the HC, GUA, and GT strains (χ^2^ = 0.22, df = 2, P>0.05; Fig 2A). No difference was observed either in female longevity between these three strains in either feeding treatments (Fed on sugar only: χ^2^ = 5.23, df = 2, P>0.05, Fig 2B; Fed on sugar and blood: χ^2^ = 3.49, df = 2, P>0.05, Fig 2C). The mean male longevity was 51.0 ± 3.1 d, 50.9 ± 3.1 d and 54.0 ± 3.1 d for the HC, GUA and GT strains, respectively. The mean longevity of females fed on sugar only was 47.85 ± 3.77 d, 44.57 ± 3.54 d and 38.57 ± 3.34 d for the HC, GUA and GT strains, respectively. The mean longevity of females fed on sugar and also offered blood meals was 47.51 ± 3.45 d, 43.14 ± 3.22 d and 40.3 ± 3.06 d for the HC, GUA and GT strains, respectively.

**Table 4:** Female fecundity (Mean ± SE) of the *Ae*.*albopictus* HC, GUA and GT strains.

**Strain**	**Replications**	**N** ^a^	**Total eggs**	**Eggs per female**
HC	3	45	2477	55.0 ± 2.3 a
GUA	3	34	1974	58.1 ± 2.5 a
GT	3	36	2042	56.7 ± 2.4 a

^a^ The number of females which laid eggs.

Within a column, values followed by different lowercase letters were statistically different (P<0.05) using ANOVA and Tukey’s post hoc test analysis.

## Discussion

The present study presents a comparative analysis of the impact of *Wolbachia* infections on the fecundity, fertility, developmental time, sex ratio, wing length and longevity of the three *Ae*. *albopictus* lines: the transinfected line HC (*w*AlbA, *w*AlbB and *w*Pip), the naturally double-infected line GUA (*w*AlbB and *w*AlbB) and the aposymbiotic line GT. The comparative analysis was performed after all the lines were backcrossed to have a highly similar genetic background (>98%) as the naturally infected GUA line which originated from the Guangzhou metropolitan area, China. Our study showed that, except for a minor impact on the wing length and immature development time, *Wolbachia* infections (double or triple) had no effect on the fitness of the *Ae*. *albopictus* lines studied.

Previous studies have reported that *Wolbachia* infections could have either a positive or negative impacton the fecundity of both fruit flies and mosquitoes. In the early 1990’s it was reported that *Wolbachia* had a negative impact on the fecundity of *D*. *simulans* [39]; however, within less than three decades, the presence of *Wolbachia* become beneficial by enhancing the host fecundity [40]. In *Ae*. *albopictus*, different studies have produced different results. For example, Islam and colleagues reported that naturally double-infected (*w*AlbA and *w*AlbB) *Ae*. *albopictus* females could produce more offspring than uninfected females [41, 42]. In contrast, the transinfected AR*w*P females of the single *w*Pip-infected *Ae*.*albopictus* strain were shown to produce fewer eggs than either naturally double-infected (*w*AlbA and *w*AlbB) or aposymbiotic females during their lifetime [22]; however, the fecundity of AR*w*P females recovered in later generations [22]. The present study found that there was no difference in the fecundity of the three *Ae*. *albopictus* lines (HC, GUA and GT).This is consistent with the other studies on the triple *Wolbachia*-infected *Ae*. *albopictus* strains HouR (*w*AlbA, *w*AlbB and *w*Ri) and two single *w*Mel-infected *Ae*. *albopictus* strains Uju.*w*Mel and HTM; however, *w*MelPop was shown to have major effects on the fitness of *Ae*. *albopictus* [31, 32, 34].

There have been reports showing that transinfected lines of insect species, for example of the Mediterranean fruit fly *Ceratitiscapitata*, exhibited a significant decrease in egg hatch rates [43, 44]. In most cases, these phenomena are usually due to incomplete rescue or the occasional failure of some eggs to maternally inherit *Wolbachia* [42, 43]. In some cases, fertility can be recovered through selection [45]. In *Ae*. *albopictus*, the triple-infected (*w*AlbA, *w*AlbB and *w*Ri) HouR line exhibited a similar egg hatch rate to the naturally double-infected (*w*AlbA and*w*AlbB) line while a reduction in egg hatch was observed in the single-infected (*w*Ri) HTR line [30, 31]. Similar patterns were unraveled with the *w*Pip-transinfected lines: no significant effect was observed in the triple *Wolbachia*-infected (*w*AlbA, *w*AlbB and *w*Pip) HC strain while the single *w*Pip-infected AP*w*P strain showed a decreased egg hatch which was recovered in later generations [22, 33]. However, the present study did not reveal any effect of the *Wolbachia* infections on the egg hatch rates either in the transinfected (*w*AlbA, *w*AlbB and *w*Pip) or in the naturally double-infected (*w*AlbA and *w*AlbB) line. All three *Ae*. *albopictus* lines (HC, GUA and GT) exhibited very high survival rates (>88%) to adulthood which is an important advantage for mass rearing and large scale applications. Previous studies showed that *Wolbachia* negatively affects survival in the immature stages of *Ae*. *albopictus*, but not the adult sex ratio [42].Our study did not reveal differences either in either trait between HC, GUA and GT strains.

The potential effect of *Wolbachia* infections on the immature development of *Ae*. *albopictus* lines has been studied previously, however the observed differences could not be clearly attributed to the symbiont [42]. Similarly, no differences were revealed in our study using the naturally double-infected GUA strain and aposymbiotic GT strain. Interestingly, however, the triple-infected HC strain developed much faster (from L_1_ to pupationand from L_1_ to adult emergence) in both females and males compared to the GUA and GT strains. In contrast, the single *Wolbachia* (*w*Pip)-infected *Ae*. *albopictus* strain AR*w*P showed no difference in development rate compared to the wild type double-infected (*w*AlbA and *w*AlbB) or the aposymbiotic strain [22]. This difference could be due to the different genetic background of the mosquito lines, the infection status or relative levels, the rearing conditions, the co-evolutionary history of these lines, or a combination of these factors. Interestingly, the shorter developmental time of the triple-infected HC strain might have a significantly positive impact on the mass rearing process by decreasing the rearing duration (by approximately half a day) and thus reducing rearing costs. In addition, this line has a more pronounced difference in the time to pupation on males than females. This might be of paramount importance for SIT and IIT applications, both of which depend on the availability of a perfect sexing system because the accidental release of females might increase pathogen transmission and certainly reduces the efficacy of the population suppression program [7, 46]. For the IIT strategy, the accidental release of transinfected females may result in population replacement, rather than population suppression [7]. However, a highly efficient sex separation method is not available for *Ae*. *albopictus* [47]. So, the additional difference observed in the development of the triple-infected HC line between two sexes could be further exploited and might provide an opportunity to solve the current lack of an efficient means of sex separation [38, 47]. An alternative approach would be to combine irradiation with IIT [7, 23, 48]. The application of low dose irradiation would sterilize the females while the released males would be fully sterile due to effects of both *Wolbachia* and irradiation [7, 33]. This combined strategy is currently being considered for the population control of *Ae*. *albopictus*, a primary dengue vector in mainland China.

Wing length is commonly used as a proxy to determine the size of adult mosquitoes. It has been shown that rearing larvae at low densities results in larger mosquitoes. Short developmental time has also been associated with large adult mosquitoes [38, 49, 50]. In contrast, our study shows that the male developmental time of the HC strain is shorter than that observed for GUA and GT strains (Table 2) and does not result in larger adult male mosquitoes (Fig 1). On the other hand, significant differences were detected in the size of adult females between the GUA and GT lines (Fig 1) despite the fact that their developmental time was similar (Table 2). Previous studies could not reveal an effect of *Wolbachia* infection on the adult size of *Ae*. *albopictus* mosquitoes [42]. Even though the present study shows that differences exist between HC and GT males (2.01 ± 0.09 mm *vs* 2.07 ± 0.11 mm), and GUA and GT females (2.60 ± 0.09 mm *vs* 2.55 ± 0.10 mm), these are essentially very minor differences and likely to be of no major importance. Although single *w*Mel-infected males were found to live longer than the wild type double-infected (*w*AlbA and *w*AlbB) or aposymbiotic males [32], most studies have not revealed any effect of *Wolbachia* infections (single, double or triple) on the longevity of *Ae*. *albopictus* males [22, 36, 41]. In corroboration, our study could not detect any effect of *Wolbachia* infection (either double or triple) on male or female longevity, irrespective of the feeding regime.

The development and application of SIT and IIT (or their combined use) for the control of *Ae*. *albopictus* depends on the mass rearing and production of high quality sterile male insects which compete with wild type males to mate with wild females in order to introduce sterility into the natural population [46, 51]. Taken all together, the presence of *Wolbachia w*Pip appears to have a minimal effect on the fitness of the artificially triple-infected (*w*AlbA, *w*AlbB and *w*Pip) HC strains, with fecundity and fertility, developmental time and longevity being comparable to the wild type *Ae*. *albopictus*. These results support the feasibility of applying mass rearing and integrated SIT/IIT to control *Ae*. *albopictus*.
